# Periampullary Diverticula Causing Recurrent Pancreatitis: A Case Report

**DOI:** 10.7759/cureus.42189

**Published:** 2023-07-20

**Authors:** Nikit Venishetty, Amish Parikh, Meesha Trivedi, Claudia Didia

**Affiliations:** 1 Department of Orthopedic Surgery, Texas Tech University Health Sciences Center El Paso Paul L. Foster School of Medicine, El Paso, USA; 2 Department of Internal Medicine, Texas Tech University Health Sciences Center El Paso Paul L. Foster School of Medicine, El Paso, USA

**Keywords:** esophagastroduodenoscopy scope, magnetic resonance cholangiopancreatography (mrcp), diverticulum, acute pancreatitis, pancreatitis causes, periampullary diverticulum

## Abstract

Acute p­ancreatitis (AP) is increasingly rising globally, especially among elderly populations. In many cases, AP can progress to chronic pancreatitis (CP) and cause damage to the pancreas. Common causes of AP include gallstones and alcoholic injury, but periampullary diverticula (PAD) have emerged as a complex etiology. PADs are rare bowel-filled outpouchings located near the main papilla or common bile duct (CBD). In this study, we present a 66-year-old female with recurrent pancreatitis that is caused by a PAD. Due to the paucity of information regarding the management of PADs, we hope this case highlights the need to advance treatment options in this area.

## Introduction

Acute pancreatitis (AP), or acute inflammation of the pancreas, has over 275,000 cases worldwide annually [1]. Its incidence has risen in the elderly population, resulting in increased severe systemic complications due to its unpredictable nature [1]. In long-standing cases, AP can evolve into chronic pancreatitis (CP), marked by permanent structural damage to and an irreversible decrease in exocrine and endocrine pancreatic function, with a reported prevalence of 0.2%-0.6% in Western countries [2]. Disease therapies for both AP and CP are relatively standard (including enteral feedings, fluid resuscitation, and analgesics). Still, the mortality rates of pancreatitis remain around 5%-10% [3,4]. The most common etiologies of AP include gallstones (40% of cases) and alcoholic injury (30% of cases), as well as rarer causes such as medication injury, acquired infection, cystic fibrosis, endoscopic retrograde cholangiopancreatography (ERCP), abdominal trauma, and pancreatic divisum [5]. Among AP's various etiologies, periampullary diverticula (PAD) has been shown to be a uniquely challenging etiology of pancreatitis [6].

PADs are defined by luminal mucosal outpouchings of the duodenum that either originate near or contain the major/main duodenal papilla (ampulla of Vater) or the intramural section of the common bile duct (CBD) [7]. PADs comprise approximately 70%-75% of all duodenal diverticula [6]. PAD's prevalence increases with age; however, the management of PADs is understudied, and there are no current guidelines [6]. We present a case of recurrent episodes of acute pancreatitis secondary to a periampullary diverticulum.

## Case presentation

A 66-year-old Hispanic female presented with a clinical history of hypertension, hypersensitivity lung disease, diabetes mellitus type 2, deep vein thrombosis (DVT), and two prior episodes of pancreatitis with severe upper abdominal pain and increased urinary frequency. She denied hematochezia, hematuria, chest pain, shortness of breath, dysuria, and any changes in bowel habits. She was afebrile with a blood pressure of 137/85 mm Hg, heart rate of 104 bpm, and respiratory rate of 18/minute. Surgical history was significant for right total knee arthroplasty, hysterectomy, and oophorectomy. Laboratory work was significant for elevated aspartate aminotransferase (AST), alanine aminotransferase (ALT), alkaline phosphatase (ALP), lipase, bilirubin, and glucose with decreased hemoglobin and hematocrit (Table 1).

**Table 1:** Complete metabolic panel and complete blood count values of the patient ALT: alanine aminotransferase, AST: aspartate aminotransferase, ALP: alkaline phosphatase, WBC: white blood cell

Serum	Patient	Reference
ALT	103 U/L	10-40 U/L
AST	65 U/L	12-38 U/L
ALP	346 U/L	25-100 U/L
Lipase	1,899 U/L	0-160 U/L
Total bilirubin	1.7 mg/dL	0.1-1 mg/dL
Total protein	5.9 g/dL	6-7.8 g/dL
Albumin	3.2 g/dL	3.5-5.5 g/dL
Glucose	178 mg/dL	70-110 mg/dL
Magnesium	1.3 mEq/L	1.5-2 mEq/L
Sodium	134 mEq/L	136-146 mEq/L
Hematologic		
Leukocyte count (WBC)	7,720/mm^3^	4,500-11,000/mm^3^
Hemoglobin	10.3 g/dL	12-16 g/dL (female)
Hematocrit	31.7%	36%-46% (female)

**Figure 1:**
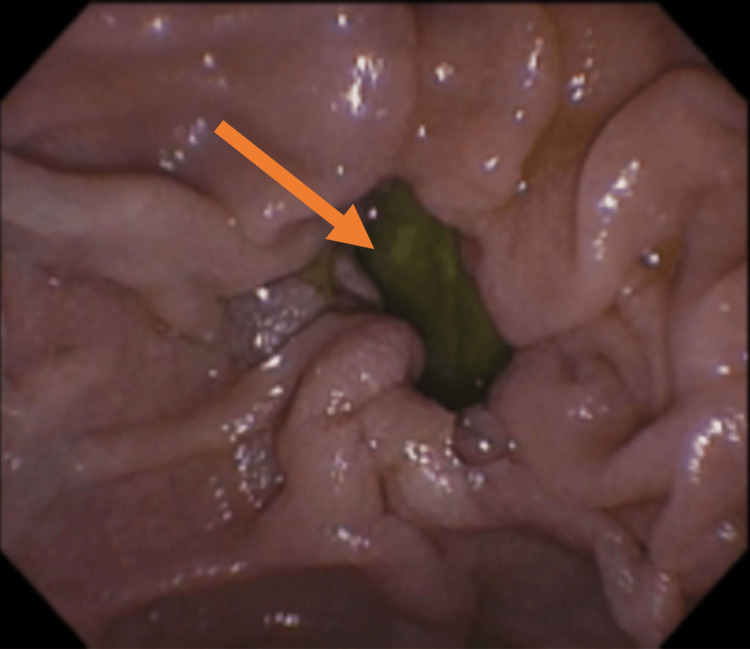
Diverticulum in the second part of the duodenum

Endoscopy revealed a small duodenal diverticulum, measuring 1 cm × 1.5 cm wide (Figure 1).

A subsequent magnetic resonance cholangiopancreatography (MRCP) showed a mildly dilated proximal CBD measuring up to 1 cm in diameter. There was no evidence of choledocholithiasis in the proximal or mid-CBD. Mild prominence of intrahepatic biliary ducts was present, but no focal mass was identified in the neck, body, or tail of the pancreas. Abdominal ultrasound displayed an unremarkable right upper quadrant.

Endoscopic retrograde cholangiopancreatography (ERCP) was then attempted, and it was discovered that the major papilla was located entirely within a diverticulum and inverted inside. Multiple attempts to cannulate and readjust the direction of the major papilla were unsuccessful using the ERCP scope and esophagogastroduodenoscopy scope or by clipping the edge of the diverticulum. After the ERCP procedure, the patient was returned to the hospital ward for ongoing care, which included intravenous fluid hydration, a clear liquid diet, laxatives (bisacodyl and miralax/polyethylene glycol), pantoprazole, and heparin for DVT prophylaxis. The patient left the hospital against medical advice before a repeat ERCP could be attempted. She later reported that her symptoms spontaneously resolved.

Six weeks later, the patient was readmitted with similar symptoms. Ultrasound showed no evidence of gallstones but showed circumferential gallbladder thickening, for which she subsequently underwent a laparoscopic cholecystectomy. This provided a resolution to further AP episodes. Given that the patient's elevated liver function tests (LFTs) and bilirubin improved concordantly with AP symptom resolution during both admissions, an obstructive etiology of pancreatitis (anatomical obstruction at the major duodenal papilla secondary to the patient's PAD, or spontaneously passed gallstone(s)) was presumed.

## Discussion

PADs have taken on more significance in the field of clinical gastroenterology, with their increased incidence likely attributable to the rising life expectancy worldwide [6]. While many of these cases are asymptomatic, patients like ours can present with severe systemic symptomatology. These recurrent episodes of pancreatitis lead to increased hospitalizations and healthcare utilization [6-8]. Treatment guidelines for PADs are not currently available but could provide more efficient treatment and reduce the number of hospitalizations in the future.

Currently, elective surgical treatment of asymptomatic or minimally symptomatic diverticula is not recommended, especially if the diverticula are found in the second part of the duodenum due to their retroperitoneal nature [6]. Symptomatic diverticula may be treated surgically or endoscopically. However, diverticulectomy procedures for abdominal discomfort are associated with high morbidity and mortality rates [6,8], and only 50% of patients with symptomatic duodenal diverticula were relieved of their symptoms when treated with a diverticulectomy in one study [8].

ERCPs for PADs are extremely difficult due to the risk of perforating the diverticulum and hemorrhage, and the success rates in previous studies are variable. The combination of ERCP paired with percutaneous transhepatic cholangiogram (TC) has shown greater efficacy in patients who are poor candidates for conventional surgical treatments. Developments of duodenoscopes with large operating channels and the "tuteur transcystique-transpappilaire" technique have demonstrated potential success for entering the duodenum without perforation; however, studies on these procedures are extremely limited [7]. Additionally, stent-guided sphincterotomy is recommended for patients who present with PADs to reduce the risk of perforation, but further studies are needed to prove its efficacy [7].

There are a range of purported mechanisms by which PADs are implicated in the pathogenesis of acute, recurrent, and chronic pancreatitis. PADs at or near the ampulla of Vater can directly obstruct biliary passage. Additionally, PADs are associated with a higher occurrence and recurrence of bile duct stones [9], and PADs may be associated with sphincter of Oddi dysfunction causing pancreaticobiliary reflux [10]. The clinical findings associated with PADs are often mistaken for biliary stones, periampullary tumors, and pancreatic pseudocysts [6], and clinicians must distinguish these pathologies for adequate management.

## Conclusions

Periampullary diverticula are extremely rare luminal outpouchings that are associated with severe complications such as abdominal pain and acute/chronic pancreatitis, as seen in this patient. There is a need for well-informed, interdisciplinary guidelines for endoscopists, surgeons, and interventional radiologists for the diagnosis of PADs (especially to distinguish PADs from classic choledocholithiasis) and the management of symptomatic PADs.
